# On Gravel, Calculus, and Gout; Chiefly an Application of Professor Liebig's Physiology to the Prevention and Cure of These Diseases

**Published:** 1843-04

**Authors:** 


### Art. XVII.

*On Gravel, Calculus, and Gout; chiefly an application of Professor Liebig's Physiology to the Prevention and Cure of these Diseases.* By Henry Bence Jones, m.a., Licentiate of the College of Physicians, and Fellow of the Chemical Society.—*London*, 1842. 8vo, pp. 142.

That diseases resulting from obvious chemical changes in the composition of the animal fluids, should be, in some degree at least, amenable to treatment based upon chemical principles, is no new doctrine; for not only is it one which would naturally suggest itself to the mind of any person acquainted with these principles, but it has been sufficiently confirmed by experience to give it a fair claim to attention. It must not be forgotten, however, that our acquaintance with the ordinary changes of composition that occur in the processes of nutrition and secretion, is at best of a very uncertain character; and, further, that we are as yet almost completely in the dark, as to the influence of the *vital* properties of the organism upon its chemical operations. It seems to us that there is too great a tendency among the chemists of the present day, to regard the human body as a mere laboratory, into which certain quantities of material are introduced, certain effects produced, and certain results obtained. We have seen a calculation of the amount of carbon expended in carrying a man to the top of Mount Blanc; by which it was demonstrated that the human machine does more work, with a less expenditure of fuel, than the best steam-engine that was ever constructed. Really such notions are scarcely one whit less absurd than those of the Iatro-chemists of the last century; indeed we very much question whether, in proportion to the prevalent intelligence of the age, and the advanced condition of physiological and chemical science, the modern absurdities are not the more glaring of the two.

Far be it from us to discourage inquiries conducted in a truly philosophical spirit; let the physiologist learn from the chemist all the facts which can throw light upon the obscurities of his science; and let the chemist push his inquiries to the utmost extent of which his objects and appliances are capable. But let not the physiologist forget, that the chemistry of the body is but a part, and a very small part, of its vital action; and let not the chemist be too ready to assume as demonstrated facts, what are merely inferences, possessing a greater or less amount of probability, from data to which a considerable uncertainty is attached.

There are few novel doctrines which command an universal assent on their first announcement. Even those which we now consider as almost self-evident axioms, have had to make their way through violent opposition. And it is scarcely to be expected that those of a less positive character should at once prevail. Indeed it would be very undesirable if it were so. All ages have witnessed the evil of undiscerning submission to commanding authority; and in modern times, more particularly, we have seen abundant instances of the prevalence of brilliant error over sober truth. The influence of great names does much; a specious aptitude or harmony on the part of the theory itself is also very effective; and when the two are combined, a very injurious result is likely to follow. But all are not so led away. There are some who are bold enough to doubt, to question, or to impugn; and these laying hold of the errors of the system, are as indiscriminating in their censure as its admirers are in its praise, and attack the whole because they find a part to be untrue. It is well for the interests of science that such objectors should exist; for error would otherwise ride as a triumphant sovereign through the domain of which truth is the legitimate owner; and it marks the beautiful adjustment of the varying tendencies of the human mind, which none but an almighty Creator could have effected, that the contest, though often protracted and severe, is sure to be rightly decided at last. *Magna est veritas, et prevalebit.*

The reception which Professor Liebig's recent work on Animal Chemistry has experienced, is an apt illustration of these tendencies. By some it has been lauded as "perfect and entire, wanting nothing," a sort of paragon, preeminent among scientific works for the accuracy of its data, the closeness of its reasoning, the comprehensiveness and originality of its principles. Everything which Liebig utters is to them law; nothing *can* be wrong, nothing even doubtful; the *ipse dixit* of the master is sufficient to establish every position; and his hypotheses are put forward as inductions, his probabilities as certainties. On the other hand, there are not wanting those who assert that his data are insecure, his reasoning frequently inconclusive, and his principles, in consequence, altogether unsound. We endeavoured, in our review of his work, to steer a middle course; satisfied that although errors might be discerned, there was much truth at the bottom; and we endeavoured, by a careful analysis, to separate the sound from the unsound parts, and to show wherein the value of the book truly lay. That our review pleased neither party, being too critical for the one, and too laudatory for the other, is a satisfactory evidence to our minds, that, whether correct or not in all its details, it was conceived and executed in a right spirit.

In the same spirit we have applied ourselves to the perusal of the work before us; in which an attempt is made to apply to pathology and therapeutics some of the physiological and chemical doctrines of Liebig, by one of his pupils and ardent admirers. The success of any such attempt must depend, in the first place, upon the truth of the doctrines themselves; and, in the second, upon the correctness of their application. To these questions, therefore, we shall now address ourselves, with, we trust we may say, a candid and inquiring spirit.

One of the most important disorders of the secreting function, is that in which an excess of lithic acid is present in the urine. This excess can only result from its presence in too large an amount, in the blood from which the product is separated; and it appears to be to the same increase, that the various phenomena of gout or arthritic inflammation are due. Hence we may regard the lithic acid diathesis as a constitutional state, characterized by an accumulation of lithic acid in the blood; and the separation of this substance through the kidneys, or its deposition in the tissues, may take place either conjointly or otherwise, according to certain peculiarities in the state of the system at the time. It is the object of the physician to prevent the undue production of lithic acid; or, to render it soluble, so that it may be excreted without difficulty. The production of lithic acid takes place, by the universal consent of physiologists and chemists, at the expense of the disintegrated albuminous or gelatinous tissues; which, having performed their appointed purpose, and lived their allotted term, undergo a change in their composition, and resolve themselves into different combinations of the same elements. For this change, the presence of oxygen is required, as it is for most of the decompositions to which the animal fabric is liable; and the requisite supply appears to be obtained through the respiratory process. Now uric or lithic acid is less highly oxygenized than urea; and it is the peculiar theory of Liebig, that the presence of an excess of lithic acid in the urine indicates a deficient oxygenation of the decomposing tissues; whilst increased oxygenation may occasion the complete conversion of uric acid into urea. He grounds this view in part upon the fact, that the urine of serpents and other reptiles, whose respiration is low, contains little or no urea, but a large quantity of uric acid; whilst the urine of the carnivorous mammalia, which lead a life of great activity, inducing active respiration, is almost destitute of uric acid, but contains in its stead a larger quantity of the highly-oxidized urea. It is a palpable objection to any argument of this kind, that birds, whose respiration surpasses that of any other vertebrata, void a large quantity of uric acid, and very little or no urea. Of this objection, Liebig attempts to dispose by remarking, that birds take very little water; and that, as uric acid is very insoluble, it cannot be so readily acted on by the oxygen, as if it were completely dissolved in a fluid. We cannot admit this explanation, however, until it shall have been shown, that the formation of urea is always preceded by that of uric acid; and that the greater or less oxidation of the latter is due simply to the mode in which it is diffused through the blood. For our own parts, we should be much more disposed to believe that the *form*, whether of uric acid or urea, is determined at the moment at which the conversion of the organized tissue into these products of secretion takes place; and that the variation in the supply of oxygen, if it have any effect at all, must be exerted upon the uric acid whilst in a nascent state. Hence we meet in birds with the anomaly of a high respiration and low oxygenation of the solid matter of the urine, a fact completely opposed to Liebig's hypothesis.

There is another objection of even greater weight, of which we do not remember that any notice has been taken by Liebig or his followers. It is this: What proof can be given that *all* the azotized matter of the urine is derived from the disintegration of the animal tissues? We are informed by Liebig that it *is* so; and he even goes so far as to state, that the amount of azotized matter in the urine may be regarded as a measure of the decomposition which takes place in the azotized tissues. But, it may be asked, how is this to be substantiated? If we suppose that no more nutriment is received into the system than is requisite to supply its wants, we can readily admit that no azotized matter will pass into the urine, until after it has performed its part in the solid structure. But if there be a surplus, either in consequence of an increase in the quantity of food, or a diminution in the demand, there would be an accumulation of nutrient materials in the blood, if means were not adopted to prevent it. Among the lower animals we apprehend that such a superabundance will rarely occur. Their natural instinct directs them to stop when they have had enough; and though a delicious *morceau* may tempt to an occasional transgression, we believe that habitual overfeeding never occurs, except among individuals which have learned the bad habits of the human species, from being domesticated with it. The gigantic boa gorges himself with an entire ox, and the gluttonous vulture distends its capacious stomach with the carrion of an almost entire carcase; but these animals are in no hurry to renew their meals, and remain in a state of slothful inactivity, until aroused to energy by the calls of hunger. Far different is the case with the lords of the creation. The gratification of the palate, not the satisfaction of the natural appetite, is too often made the object of desire; and instead of working off, by exercise and exposure, the ill effects of the repletion, a life of inactivity and indulgence is preferred. A heavy reckoning is thus prepared for a subsequent day; and who shall say that the punishment is not deserved? If a larger quantity of non-azotized matter is taken into the system than the liver and lungs can dispose of, it is deposited as fat. But what becomes of the azotized substances? They do not go to swell the bulk of the muscles. On the contrary, the nutrition of the muscular structure is augmented by the very cause that produces its disintegration. The effect of exercise in increasing the development of the muscular tissue is familiar to every one; and thus it serves, in two ways, to prevent the accumulation of azotized substances in the blood. But if high-feeding and inactive habits be combined, will Professor Liebig tell us how these substances are disposed of, except by passing,in an altered condition, into the urine? It is very possible that, in consequence of primary mal-assimilation (as Dr. Prout calls it), resulting from disordered state of the digestive organs, these matters *never could* have been appropriated by the tissues, and that they must hence either accumulate in the blood or be separated by a secreting process. But we cannot see how the simple excess of truly assimilable matter can be disposed of, except in a similar mode. We believe that the fibrin of the blood, like the solid tissues, has a limited term of existence *as such;* and that it must either be converted into solid tissue, or must undergo a change of composition. In the healthy state of the system, there is a constant demand for as much fibrin as the blood supplies, and there is consequently no waste; but if the supply is greater than the demand, how can the excess be removed, save through the excretory organs especially concerned in carrying off the superfluous and effete azotized matters of the system, namely, the kidney and the skin?

We have stated these objections, not in a carping spirit of detraction, but with the simple desire to arrive at the truth. We took occasion to point out, on af ormer occasion, (Vol. XIV, p. 514,) the exclusiveness of Liebig's views on the connexion of the biliary and pulmonary secretions, and their complete inapplicability to classes of animals formed on a plan different from that which he had considered; and it appears to us that he has fallen into a similar error in the present instance, from reasoning upon one particular state of the nutritive functions,—that which, unfortunately, is not of very frequent occurrence in man,—the exact conformity of the supply of food to the demand existing in the system. As will presently appear, we are not indisposed to admit the validity of Professor Liebig's hypothesis, as a *partial* explanation of the lithic acid diathesis; but we feel assured that many other causes may act, either concurrently or separately, in its production.

We are obliged to Dr. Golding Bird (Medical Gazette, Feb. 25, 1843,) for having directed attention to the important test of the validity of Liebig's theory, which is afforded by M. Edmund Becquerel's numerous analyses of the urine in various diseases. If this theory were correct, we ought to find that, in diseases in which there is deficient oxygenation of the tissues, the proportion of urea will be diminished, and that of uric acid will be increased; whilst in phthisis and in inflammatory diseases, in which, according to Liebig, there is an excessive action of oxygen on the tissues, the uric acid should almost or altogether disappear from the urine, being converted into urea. That this is far from being the case, the subjoined table will show.

| Disease. | *Quantity in 24 hours of* Uric Acid. | *Quantity in 24 hours of* Urea. | *Proportion of Uric Acid to Urea.* |
|---|---|---|---|
| | Grains. | Grains. | |
| Healthy urine (general average) | 8·1 | 255 | 1 : 30·37 |
| Chlorosis, minimum of five cases | 1·8 | 77·5 | 1 : 43 |
| Chlorosis, maximum of five cases | 6·0 | 172 | 1 : 29 |
| Pulmonary emphysema with extreme dyspnœa... | 4·9 | 172 | 1 : 35·1 |
| Phthisis, tubercles softened | 9·1 | 66·7 | 1 : 7·33 |
| Phthisis, three days before death | 9·8 | 29·4 | 1 : 3 |
| Morbus cordis, with jaundice | 9·82 | 73·3 | 1 : 7·6 |
| Acute hepatitis, with jaundice | 11·18 | 61·6 | 1 : 5·6 |
| Jaundice | 17·75 | 285·6 | 1 : 16·1 |
| Milk-fever | 19 | 133 | 1 : 7·47 |

In chlorosis, a disease marked by the deficiency of the red corpuscles, and consequent lowness of oxygenation, we find that, whilst the whole amount of azotized matter in the urine was greatly diminished, the uric acid had undergone a special decrease; instead of being greatly increased, either absolutely, or at any rate proportionally to the urea, as Liebig's hypothesis would predicate. There is scarcely a better example of prolonged and distressing dyspnea, almost reducing the body to the imperfectly-oxygenated state of that of the reptile, than is afforded by a severe case of pulmonary emphysema. It might be expected in such a case, that there would be no oxygen to spare for the production of urea, but that the whole of the azotized matter would be thrown off in the state of urate of ammonia, as in reptiles. So far is this from being the case, that the quantity of uric acid is not only diminished to little more than one half its usual amount, but it is diminished relatively to the urea present in the same urine. Again, in phthisis we observe that the whole azotized matter of the urine is very small in amount; and there would, therefore, be still greater reason to imagine that, by the excessive action of oxygen upon the tissues (which, we must observe, appears to us but a mere vague hypothesis,) even with a diminished respiration, the uric acid should all be converted into urea. But so far is this from being the case, that, with a decrease of the urea to about one fourth (in one case,) or one eighth (in the more advanced case,) of the natural amount, there is an actual increase in the quantity of uric acid; so that its proportion to the urea, instead of being diminished, is increased from 1 in 30·37, to 1 in 7·33, and even to 1 in 3. A corresponding increase is seen in various inflammatory diseases: and we fully agree with Dr. Golding Bird in the belief, that a theory cannot be true, which, when brought to the test of observation, affords results so entirely at variance with its predictions.

The theory of Liebig overlooks, also, several other causes of the appearance of lithic acid deposits in the urine; each of which may act occasionally, either alone or in combination with others. Thus it is now generally admitted that the lithic acid, when held in solution, is in combination with ammonia or some other base; since any other acid which detaches that base from it, causes its precipitation. Now this may be accomplished by the formation of an organic acid in the system itself, as well as by artificially adding it as a precipitant. In certain forms of dyspepsia, there is an excessive production of lactic and even of muriatic acid in the stomach; and with these conditions, we very commonly find united a lithic acid deposit in the urine. The acids generated in the stomach, combine with the ammonia that would otherwise have held the lithic acid in solution; and the latter consequently falls as a gravelly sediment. The same effect may be produced by ingesting a mineral acid. An important therapeutic direction is hence indicated ; that, in the choice of articles of food, we should be careful to avoid those which are most liable to contribute to the generation of acids in the stomach. We need not inform our readers of the practical importance of this principle, when tried by the test of experience.

Any interruption to the secretory action of the skin, also, may produce a deposit of lithic acid ; and this perhaps, in various ways. The skin, besides discharging a large quantity of water, throws off no less than 100 grains of azotized matter daily, and its function is of great importance in the removal of such products. Now if perspiration be checked, there will be an additional amount to be thrown off by the kidneys ; and this may take the form of lithic acid. Again, checked perspiration will occasion an undue accumulation of lactic acid in the system; and this, combining with the ammonia, causes a separation and precipitation of lithic acid in the urine. Dr. Bence Jones suggests, in the treatise before us, another mode in which the accumulation of lactic acid may produce a lithic acid deposit; he regards, with Liebig, the lactic acid as one of the substances which is got rid of, in the form of carbonic acid and water, by the respiratory function, which introduces oxygen to carry it off. Hence he regards it as capable, by its superior affinity for oxygen, of preventing the operation of this oxygen upon the uric acid, by which it would be converted into urea. But this explanation rests upon the hypothesis, respecting the dependence of this change upon the amount of oxygen taken by respiration, which we have already examined and found insufficient. At any rate, we have other explanations of a more satisfactory character, to account for the well-known connexion between the lithic acid deposit and obstructed perspiration.

In regard to the metamorphosis of the albuminous tissues, Dr. Jones adheres to the theory of Liebig ; who attempted to show, as our readers will remember, that their constituents are at once resolvable into the essential constituents of the biliary and urinary excretions, namely, choleic acid and urate of ammonia. Dr. Jones endeavours to extend the same kind of reasoning to the gelatinous tissues. We are astonished to find him asserting (p. 12,) that the deposit of urate of soda in gouty concretions iu the neighbourhood of the joints, *demonstrates* that the formation of uric acid takes place at the expense of the gelatinous tissues in which the deposits occur. In our humble judgment, this is no more demonstrated, than it is that the deposit of lithic acid in the urine proves that this acid results from the disintegration of the tissue of the kidney. Dr. Jones must be very ignorant of physiology, if he imagine that the deposit of a certain substance in any particular situation indicates anything else, than that the substance pre-existed in the blood, and that it is separated from it by some local action. Abundant proof of this is afforded by the operation of numerous remedial and poisonous agents. It appears to us that, following the plan which has been adopted by Dr. Jones in disposing of the elements of the gelatinous tissues, we need have no difficulty in theoretically metamorphosing any substance into any other. It is only to add or subtract a few atoms of oxygen, hydrogen, carbon, or nitrogen (which always seem to be most conveniently at hand for the purpose,) to turn almost any organic substance into any other. Thus according to Dr. Jones, "the formula which Prof. Liebig gives, as corresponding most nearly to the analysis of gelatine is 32 c., 5 n., 27 h., 12 o.; deducting from this, one equivalent of urate of ammonia, 10 c., 5 n., 7 h., 6 o., there remains 22 c., 20 h., 6 o. But the formula for fat is 22 c., 20 h., 20 o. So that we may suppose that gelatine, by giving up four atoms of oxygen, might be converted into urate of ammonia and fat." (p. 14.) Another solution is afterwards given. "Gelatine, with the addition of four equivalents of water and two of oxygen, may be converted into fat, ammonia, urea, and carbonic acid; and the occurrence of fat in the cellular tissue may perhaps be regarded as rendering this view more probable." (p. 16.) By the same convenient process, all the chemical transformations in the body may be most readily explained; nothing is required but a table of the formulæ of the various proximate principles, and a competent knowledge of the rules of addition and subtraction. But it unfortunately happens, that a dozen or two of such explanations, differing widely from each other, might be framed by any ingenious person: and it might not be easy to pitch upon the right one.

We would not be understood as objecting to the application of chemical reasoning in explaining these transformations; but merely to this random mode, which we fear may become too prevalent, in consequence of the real or supposed sanction given to it by the highest authority. Every person, in the least degree acquainted with the principles of logic, is well aware that, in all inferential or deductive reasoning, the liability to error increases in a very rapid ratio, at every step involving the least uncertainty. Where the data are insecure, and a moderate degree only of probability attends two or three subsequent stages of the reasoning, the chances are very much *against*, rather than in favour of, the correctness of the conclusion to which they lead. It seems to us that Dr. Jones has forgotten this important principle; and has reasoned upon that which *may be*, as if it actually *were*. He commences his fourth chapter in the following manner: "It has been shown that uric acid may be produced from the gelatinous as well as from the albuminous tissues; that from the changes which take place in the tissues, uric acid may be formed." Now we would not be understood as denying the truth of the position. It may or may not be correct; but we simply say that Dr. Jones has not proved it, or even rendered it probable. His only argument is derived from the deposition of the urates in the gelatinous tissues surrounding the joints, of which we have already shown the fallacy; and from the *possible* transformation of gelatine into uric acid, by the subtraction of certain elements. We would undertake, upon the same plan, to convert (theoretically) any azotized substances into uric acid; and it would only require the addition of a few atoms of ammonia, and other small alterations of the like nature, to elaborate uric acid, *on paper*, out of starch or sugar. But until this or any other transformation has been shown to be possible, in the laboratory of the chemist, we shall not regard it as proved to take place in the living body.

For the discussion of details of this kind, however, it is not requisite that we should diverge further from the main question—the source of the over-production of lithic acid in the body, and the mode in which it may be prevented. Considering with Liebig, that the whole of this acid existing in the urine is the product of the decomposition of the tissues, Dr.

Jones proceeds to inquire into the conditions of its undue appearance. He supposes that, when an increased amount of decomposition of the tissues occurs, in consequence of the excessive action of oxygen upon them, no additional excretion of uric acid will take place; since the supply of oxygen is sufficient to convert it all into urea. The increase in the proportion of uric acid he attributes solely to the deficient action of oxygen; so that the change which ought to take place within the body, does not occur. The following quotation will exhibit his views. He states it as a general law, that "the quantity of uric acid which is thrown out of the body varies inversely with the quantity which undergoes a change in the body;" and thus continues:

"On looking to the agents which effect this change, it is seen that oxygen is the most absolutely necessary, and that the other agents, the heat, water, and alkali, are chiefly useful, inasmuch as they enable and promote the action of the oxygen on the uric acid in the body. Thence it may be concluded, that the quantity of uric acid which is thrown out varies inversely with the amount of action of the oxygen; and thus the problem of the cure of the uric acid diathesis depends, tonics being excepted, on the question, how the uric acid in the body can be most acted on by oxygen, how it can be changed into urea and carbonic acid. It appears that this may be done—

"1st. By giving a large supply of oxygen, as by exercise, by cold air, and by medicine, as nitrous oxide water and iron.

"2d. By diminishing the quantity of other substances, on which the oxygen acts more readily than on the uric acid; that is, substances consisting of hydrogen, carbon, and oxygen only; as by abstaining from these as food, by removing them by aperients, and by sudorifics.

"3d. By keeping all the uric acid which is produced, in solution, by water, and by alkalies." p. 23.

The increase of oxygenation resulting from exercise and from cold, has already been remarked upon in our review of Liebig's work. That supposed to be produced by nitrous oxide water is hypothetical. That occcasioned by the administration of iron is much more certain. We know that, whether the iron they contain be or be not the chief instrument of their efficacy, the red corpuscles are the principal carriers of oxygen from the lungs to the systemic capillaries; and we also know that a decided increase in their number takes place, during the administration of iron, in anemic states of the system. In one of the cases of chlorosis mentioned by Andral, the proportion of red corpuscles had sunk from 127 in 1000, to no more than 49·6; and, after the administration of iron for a short time, it was found to have risen to 64·3. In another case, in which the remedy had been longer persevered in, the increase was from 46·6 to 95·7, or more than double. Thus, then, it will be very easy for Dr. Jones to verify his reasoning by an appeal to experience, and we should recommend him to do so before he again comes before the public to direct the treatment of disease on chemical principles. As we have already pointed out, the *proportion* of lithic acid in chlorosis is *less* instead of *more* than that which is found in health; and the *absolute* quantity is very far less. The contrary would be the result of Dr. Jones's theory; and the return to the natural standard would be coincident with the multiplication of red corpuscles. As chlorotic patients are so very easily to be met with, we trust it will not be long ere Dr. Jones furnishes us with an account of his experience on this point. We should add that he attributes to iron and similar tonics the effect of "rendering the vital powers more capable of resisting the action of the oxygen on the tissues of the body."

On the second class of measures for occasioning the oxygenation of the uric acid, Dr. Jones insists strongly; and we shall hereafter see, that he makes it the foundation of the regimen he considers applicable to the treatment of gout and gravel. The non-azotized substances, which predominate in most forms of vegetable food, and which do not serve (according to Liebig) for the nutrition of the albuminous or gelatinous tissues of animals, have a strong affinity for oxygen; and if they are present in the fluids in too large an amount, they will become oxidized in preference to the uric acid, and will thus prevent it from being converted into urea. These are chiefly thrown off by the respiratory apparatus, when in an oxidized condition; in the secretion of the liver and intestinal glands, on the other hand, there is little or no addition to their natural proportion of oxygen. Hence, according to Dr. Jones, we may diminish the proportion of these readily oxidizable substances in the blood, not only by stopping the supply, but by causing an increase of secretion from the liver and intestinal glands through the action of purgatives, and especially of mercurials. These medicines will have the further effect of causing the secreted products to be discharged from the intestinal canal, instead of remaining to undergo partial re-absorption and oxidation, as is supposed by Liebig to be the case in regard to the chief elements of the bile. It is upon this principle that Dr. Jones explains the beneficial action of medicines which promote the secretion from the liver, in the treatment of gout; and we have little doubt that it may be correct as far as it goes. The quantity of these non-azotized substances may be further reduced by emetics which produce bilious vomiting; and by sudorifics. In regard to this last class of medicines Dr. Jones remarks, that "the great benefit which is occasionally experienced from the sudorific medicines is not thus, perhaps, sufficiently accounted for; because the quantity of these substances which can be removed by perspiration cannot be supposed to be very great." *We* find no difficulty in accounting for it by the fact, altogether overlooked by Dr. Jones, that a large quantity of azotized matter, not very unlike urea, is excreted from the skin; and the quantity left to be thrown off by the kidneys is therefore just so much less.

With regard to the third indication, Dr. Jones remarks that the effect of an increased supply of fluid is not confined to dissolving the excess of urate of ammonia, which would otherwise be deposited as a sediment; but that it also favours the further oxygenation of the uric acid. This is, we presume, the idea on which Liebig would explain the presence of urate of ammonia, almost to the exclusion of urea, in the urine of birds.

We now come to the application of these principles, in the cure or mitigation of the uric acid diathesis. We are by no means disposed to deny their partial correctness; but we do not think that the disordered state in question is by any means sufficiently accounted for upon the hypothesis of Liebig; and if this be adopted in the exclusive manner in which Dr. Jones has set it forth, it cannot but lead, in our opinion, to great errors in practice. There are many points of treatment, regarding which there cannot be much question, whatever hypothesis be adopted; such are the propriety of exercise, pushed to as great an extent as may be, without such fatigue as shall permanently depress the vital powers; and abstinence from superfluous sleep. These measures are insisted on by Dr. Jones, as augmenting the quantity of oxygen introduced into the blood; but their acknowledged efficacy may be equally well explained in another way. For if it be supposed that, in man, the uric acid is chiefly formed at the expense of the *superfluous* azotized compounds in the blood,—then active exercise, and abstinence from unnecessary sleep, tend to draw off these substances, by converting them into newly-organized tissue, the product of whose decomposition may be urea. The most important rule, however, is the one on which the greatest question is likely to be raised,—the proper diet to be adopted. It is well known that much success has attended the treatment of the lithic acid diathesis, by a diet, containing but a small proportion of albuminous ingredients, and a large proportion of farinaceous matter; a measure suggested by the very obvious idea, that the stoppage of the supply would be likely to induce a diminution of the product. On the other hand, many authorities have maintained that more benefit is derivable from a moderate diet in which animal flesh shall greatly predominate. Now we cannot help imagining that there has been a want of discrimination in regard to the causes of the uric acid deposit; and that one or the other kind of diet may be preferable in particular cases. That the disease may be brought on by the simple excess of mal-assimilated albuminous matter, resulting from habitual indulgence in an excess of food, we think that common experience fully proves; and, from the considerations already urged, (p. 504,) we cannot but regard it as probable, that the superfluous azotized matter ingested, passes off in the form of urate of ammonia, without becoming part of the tissues at all. What remedy can be thought of as so likely to be successful in such cases, as a diminished ingestion of albuminous matter, so as to afford no more than the necessary supply to the tissues? We fearlessly appeal to the test of experience in this matter. Who, as Dr. Graves well remarks, ever heard of a case of gout amongst the potato-eating peasantry of Ireland? Who, we will add, ever heard of one among the oatmeal-feeders of Scotland? We will venture to affirm that there is an equal rarity amongst the rice-feeding Hindoos, low as is their oxygenation, in consequence of the warmth of the surrounding medium. Every practitioner must have met with examples, in which a simple reduction in the quantity of animal food ingested, has caused an immediate disappearance of the lithic acid from the urine. Sometimes it is necessary to push this reduction to a still greater extent; thus, we have known an instance in which no decided benefit was obtained, until the patient was restricted to the Hindoo diet of rice and capsicums, which produced a complete cure. Upon Dr. Jones's principles, this ought to have produced the most serious aggravation; for the diet which he recommends, is one from which starch and other non-azotized substances are almost completely excluded.

But we are well aware that such cases form only a part of those, in which lithic acid deposits appear in the urine. Of these, some are clearly attributable to the excessive production of lactic acid in the stomach; and in these, such a diet as the one just mentioned would be extremely prejudicial, on account of the tendency of farinaceous and saccharine matter to be converted into this acid. In cases of this kind, it is doubtless of service to exclude such substances almost completely; and to select those which are least liable to undergo conversion into lactic acid. The same diet would doubtless be useful in cases, if such there be, to which the hypothesis of Liebig is applicable; since a superfluity of non-azotized matter in the blood will necessarily occasion a withdrawal of a part of the oxygen for their oxidation. But it must be remembered that, upon Liebig's own showing, the best diet for man is one in which sufficient non-azotized matter is ingested, to supply what is required for respiration; since otherwise, it would be necessary to eat a very much larger amount of animal flesh, to afford the carbon requisite for the process. Any such increase would provide more work for the kidneys, and would therefore be injurious. Our principle would be, to regulate the diet in such a manner, that the patient shall take in just enough, whether of azotized or non-azotized materials, to supply the wants of his system, and no more. This is the direction of Sir W. Temple, himself a sufferer: "Simple diet, limited by every man's experience to his own easy digestion, and thereby proportioning, as near as well can be, the daily repairs to the daily decays of our wasting system." A part of the difference of opinion in regard to the results of a sparingly-azotized diet, may be due to the fact, that it is least likely to succeed in those whose digestive powers are enfeebled by a long course of excess, and by repeated attacks of the disease to which it leads,—in consequence of the difficulty which such persons have in the digestion of most vegetable substances. We believe that if a comparatively young and vigorous person, suffering it may be through hereditary tendency to this disorder, will rigorously adhere to a diet as little azotized as possible, and will combine this with exercise, and other simple prophylactic measures, he may keep himself free from the recurrence of it. Few of the auditors of the late Dr. James Gregory, of Edinburgh, will have any doubt on this point.

Respecting the operation of purgatives and sudorifics, Dr. Jones's theory has already been given. He considers them peculiarly efficacious in gout, in which, according to Liebig, the excess of *non-azotized* compounds in the blood, abstracting the oxygen required for the conversion of the uric acid into urea, is the essential character of the disease. When Dr. Jones has shown us what becomes of the azotized matter in the body of a man who eats twice as much animal matter as his inert system requires for its reparation, we may begin to open our understandings to this theory; but at present they are obscured by a cloud which our unaided common sense cannot disperse. If this were the true explanation, gout would be one of the commonest diseases of the ill-fed labouring population, and comparatively rare in the luxurious aristocrat.

We shall not follow Dr. Jones into any further detail upon this part of the subject; since he does not offer any new therapeutic indications, but confines himself to explaining the *modus operandi* of those of acknowledged efficacy. This, we think, may be made to suit principles of a different character, at least equally well.

The Oxalic Acid Diathesis is the next subject treated of; and as Dr. Jones's notice of it is merely an expansion of Liebig's doctrine, that a partial oxidation of uric acid, not sufficient to produce urea, may generate oxalic and carbonic acids, we need not detain our readers by dwelling upon it. We may remark, however, that this principle, if correct, is of great importance, as leading to a much more rational plan of treating this diathesis than any before adopted. *This* conversion is not a theoretical one, but may be effected in the laboratory; and the frequent alternation of oxalic and uric acids in calculi is a pretty evident proof of their close chemical relation. The treatment of this diathesis will be the same, therefore, as that which is found to be satisfactory in curing the uric acid diathesis; and we need not attach any importance to abstinence from saccharine substances, which were formerly considered as the source of it, since there is no evidence whatever that oxalic acid is ever formed from sugar in the living body.

In the chapter on the Phosphatic Diathesis we do not find anything peculiarly worthy of note; except that Dr. Jones lays much stress on the avoidance of articles of food which contain phosphorus or the phosphates in large amount, and shows from late researches that these substances are much more generally diffused through organized products than is usually supposed.

The remainder of Dr. Jones's treatise consists of a summary of the different characters of calculi, and an account of the attempts that have been made at various times to get rid of them by the action of solvents. In this we find nothing worthy of note; and, having already occupied the space permitted to us, with the discussion of what we regard as the leading principle or *idea* of the book, we shall close it with a word of friendly advice to its author, and to other young chemists who may be led to commit themselves in a similar manner to the captivating novelties of an ingenious theory. No one can reasonably object to their *adoption* of principles put forward by so eminent and commanding an authority as that of Liebig; but they should remember that these principles are confessedly theoretical; and we feel sure that their author would be averse to the deduction of rules of practice from them, until they have been tested by more extensive examination, both chemical and physiological. We hold that any one who comes forward to instruct the public in any new mode of practice, especially one that is founded upon abstract principles, is bound to test the correctness of his views in every possible way, before he lays down, *ex cathedrâ*, the rules of treatment which the practitioner is to follow. For what is the result, if he comes forward, as we think Dr. Jones has done, too hastily? The book falls into the hands of a number of persons quite unqualified to judge of the correctness of the principle on which the rules of practice are founded; they have heard from high authorities that Liebig is next to infallible; and, startled at the array of incomprehensible symbols which the theoretical discussion presents, they turn to the practical portion, feeling sure that a pupil of the great Maestro, interpreting his doctrines, *must* be right, and delighted at the prospect of having some plain and simple code by which they may be governed in the treatment of this perplexing class of disorders. It is on this account that we have thought it our duty to criticise the work with peculiar attention. We have bestowed no slight amount of time and thought upon it, that we might not be open to the imputation of having hastily disposed of the first application of Liebig's physiological doctrines to the science of therapeutics. But we must add, that we should have been saved a great deal of trouble, had our author's train of reasoning been more clearly expressed, or his style less faulty. The sentences are long and involved, and their grammatical construction is often extremely defective. In proof of this we refer the reader to the second paragraph in p. 8, and the latter half of p. 10.

